# The Impact of Transcranial Direct Current Stimulation on Upper-Limb Motor Performance in Healthy Adults: A Systematic Review and Meta-Analysis

**DOI:** 10.3389/fnins.2019.01213

**Published:** 2019-11-15

**Authors:** Ronak Patel, James Ashcroft, Ashish Patel, Hutan Ashrafian, Adam J. Woods, Harsimrat Singh, Ara Darzi, Daniel Richard Leff

**Affiliations:** ^1^Department of Surgery & Cancer, Imperial College London, London, United Kingdom; ^2^Department of Clinical and Health Psychology, Center for Cognitive Aging and Memory, McKnight Brain Institute, University of Florida, Gainesville, FL, United States

**Keywords:** transcranial direct-current stimulation (tDCS), systematic review, meta-analysis, motor, healthy, performance

## Abstract

**Background:** Transcranial direct current stimulation (tDCS) has previously been reported to improve facets of upper limb motor performance such as accuracy and strength. However, the magnitude of motor performance improvement has not been reviewed by contemporaneous systematic review or meta-analysis of sham vs. active tDCS.

**Objective:** To systematically review and meta-analyse the existing evidence regarding the benefits of tDCS on upper limb motor performance in healthy adults.

**Methods:** A systematic search was conducted to obtain relevant articles from three databases (MEDLINE, EMBASE, and PsycINFO) yielding 3,200 abstracts. Following independent assessment by two reviewers, a total of 86 articles were included for review, of which 37 were deemed suitable for meta-analysis.

**Results:** Meta-analyses were performed for four outcome measures, namely: reaction time (RT), execution time (ET), time to task failure (TTF), and force. Further qualitative review was performed for accuracy and error. Statistically significant improvements in RT (effect size −0.01; 95% CI −0.02 to 0.001, *p* = 0.03) and ET (effect size −0.03; 95% CI −0.05 to −0.01, *p* = 0.017) were demonstrated compared to sham. In exercise tasks, increased force (effect size 0.10; 95% CI 0.08 to 0.13, *p* < 0.001) and a trend towards improved TTF was also observed.

**Conclusions:** This meta-analysis provides evidence attesting to the impact of tDCS on upper limb motor performance in healthy adults. Improved performance is demonstrable in reaction time, task completion time, elbow flexion tasks and accuracy. Considerable heterogeneity exists amongst the literature, further confirming the need for a standardised approach to reporting tDCS studies.

## Introduction

Transcranial Direct Current Stimulation (tDCS) is a non-invasive method of brain stimulation proposed to have beneficial effects in both cognitive and motor domains. Benefits have been demonstrated in patients with chronic pain syndromes (Fregni et al., 2006; Fenton et al., 2009; Fagerlund et al., 2015) and neuropsychiatric conditions (Baker et al., 2010; Loo et al., 2012; Palm et al., 2012; Kaski et al., 2014; Bandeira et al., 2016; Breitling et al., 2016), whilst in the healthy population, there is increasing scientific interest in the motor enhancing properties of the technology. Aligning with this trend, an increasing number of commercial companies (Edwards et al., 2017) promote the augmentation of motor abilities with tDCS including greater muscular power output (Okano et al., 2015; Huang et al., 2019), longer athletic endurance (Vitor-Costa et al., 2015; Park et al., 2019) and improved posture and balance (Kaminski et al., 2016; Saruco et al., 2017). This arena is most commonly explored through anodal tDCS to the primary motor cortex (M1), although the precise mechanism of action remains a matter of debate (Giordano et al., 2017). Excitability changes within M1 have been demonstrated, as evidenced through an increase in size of motor evoked potentials within the small muscles of the hand (Nitsche and Paulus, 2000, 2001). Similarly, tDCS transiently modulates cortical activation by raising the resting membrane potential of neurons closer to the activation threshold, thus increasing neuronal excitability (Bindman et al., 1964; Nitsche and Paulus, 2000). These neurophysiological changes persist after stimulation and are suggested to be associated with upregulation in N-methyl-d-aspartate receptor activation (Liebetanz et al., 2002). Regardless of these neurophysiological findings, there is a lack of consensus on the impact of tDCS on motor function in healthy individuals.

Despite a recent surge in meta-analyses on the effect of tDCS on aspects of cognitive function (Medina and Cason, 2017; Nilsson et al., 2017; Westwood and Romani, 2017; Simonsmeier et al., 2018), efforts to quantify the impact on motor function in healthy individuals are few in number (Bastani and Jaberzadeh, 2012; Hashemirad et al., 2016; Machado et al., 2019). Notably, Bastani and Jaberzadeh (2012) performed a meta-analysis focusing on motor cortex excitability and motor function but only included two studies involving healthy participants. Subsequently, Hashemirad et al. (2016) observed that multiple tDCS sessions over M1 induced significant task improvement but this review was limited to motor sequence learning. Other narrative reviews have summarized the effects of tDCS on motor tasks in healthy individuals with enhancing effects demonstrated in bimanual motor skills (Pixa and Pollok, 2018), motor learning (Reis and Fritsch, 2011; Buch et al., 2017), and exercise performance (Angius et al., 2017).

Whilst prior reviews (Reis and Fritsch, 2011; Angius et al., 2017; Buch et al., 2017; Pixa and Pollok, 2018) provide valuable summaries of tDCS studies, a meta-analysis would confer more critical and robust assessment of the impact of tDCS on motor function. Firstly, meta-analysis better estimates the effects that exist within the target population rather than limited to individual studies. Secondly, precision and accuracy of effect sizes is improved through pooled data offering greater statistical power than smaller separate sample sizes. Furthermore, it facilitates identification of methodological patterns or variables that could contribute to conclusions or, similarly, identify inconsistencies that lead to discrepancies within findings.

To date, there has been no systematic evaluation and meta-analysis of the overall impact of tDCS on upper limb motor performance in healthy adults and this paper aims to provide an up-to-date comprehensive analysis of available literature in this regard.

## Methods

### Search Strategy

A comprehensive electronic search (Appendix 1), of three databases was conducted, namely: (a) MEDLINE (1946—August 2018), (b) PsycINFO (1806—August 2018), and (c) EMBASE (1947—August 2018). Due to variability in motor tasks and outcomes in tDCS literature, the search initially identified all randomised-controlled trials involving tDCS. Additional studies were gathered from cross-referencing bibliographies of included papers and from Google Scholar. The date of the last search conducted was 01 August 2018.

### Eligibility Criteria

Retrieved articles were only included if they met the following inclusion criteria:

1. Studies performed on healthy subjects.
2. Studies requiring subjects to perform a motor task involving the upper limbs
3. Studies with published outcome variable data (raw or summary statistics)
4. Sham-controlled studies.

Reviews, case reports, letters, opinions, and conference abstracts were not included. Studies were limited to those carried out on adult human subjects and reported in English language. Any studies using subjects with prior expertise in tasks were not included e.g., pianists in finger tapping tasks or strength-trained athletes in elbow flexion tasks. Any studies which utilized additional interventions alongside tDCS, including pharmacological or other neuro-interventions (e.g., Transcranial Magnetic Stimulation), were also excluded.

### Data Extraction

Titles and abstracts of all retrieved articles were screened by three of the reviewers (RP, JA, and AP) to identify relevant studies. Relevant articles that met inclusion criteria were obtained in full text and further assessed for eligibility by the same authors. Any disagreements during the selection process were resolved by discussion with a fourth, senior author (HA). Final selected studies are summarized in Table 1.

**Table 1:** Characteristics of studies selected for pooled statistical analysis.

**References**	**Sample size**	**Stimulation**	**Reference**	**Current (mA)**	**Current Density (mA/cm^**2**^)**	**Duration (min)**	**Task**	**Outcome measure used in pooled analysis**
Apšvalka et al. (2018)	50	R M1	C-SOR	1	0.029	20	Finger sequence	RT and ET (s)
Arias et al. (2016)	13	L M1	R M1	1	0.029	10	Visuomotor adaptation	RT (ms)
Carlsen et al. (2015)	17	SMA (A+C)	Forehead	1	0.123	10	Simple reaction time task	RT (ms)
Dumel et al. (2016)	23	L M1	C-SOR	2	0.044	20	Serial reaction time task	RT (ms)
Ehsani et al. (2016)	39	L M1; cerebellum	R SOR; R arm	2	0.080	20	Serial reaction time task	RT (s)
Focke et al. (2017)	36	L PMC (A+C)	C-SOR	0.25	0.029	10	Serial reaction time task	RT (ms)
Galea et al. (2011)	40	L M1; R cerebellum	C-SOR; R Buccinator	2	0.080	15	Visuomotor adaptation	RT (ms)
Heise et al. (2014)	32	L M1	C-SOR	1	0.040	20	Serial reaction time task	RT (ms)
Horvath et al. (2016)	230	L M1 (A+C)	C-SOR; R M1, R wrist	1; 2	0.029; 0.057	20	Serial reaction time task	RT (ms)
Kang and Paik (2011)	11	L M1	C-SOR, R M1	2	0.080	20	Serial reaction time task	RT (ms)
Kantak et al. (2012)	13	R M1, PMC	C-SOR	1	0.125	15	Finger sequence	RT (s)
Karok and Witney (2013)	20	R M1	C-SOR; L M1	1.5	0.060	10	Serial finger tapping	RT (s)
Samaei et al. (2017)	30	Cerebellum	R Shoulder	2	0.080	20	Serial reaction time task	RT (s)
Shimizu et al. (2017)	45	Cerebellum (A+C)	Buccinator	2	0.057	20	Serial reaction time task	RT (s)
Waters-Metenier et al. (2014)	52	R M1	L M1	2	0.057	25	Configuration task	RT and ET (s)
Boggio et al. (2006)	8	R M1; L M1	C-SOR	1	0.029	20	JHFT	ET (s)
Convento et al. (2014)	12	R M1; L M1; R PPC; L PPC	C-SOR	2	0.080	10	JHFT	ET (s)
Doppelmayr et al. (2016)	83	L M1; cerebellum; R parietal	HD montage	1	0.318	21	Visuo-motor task	ET (s)
Hummel et al. (2010)	10	R M1	C-SOR	1	0.040	20	JHFT	ET (s)
Karok et al. (2017)	30	R M1	L M1, C-SOR	1.5	0.060	15	Purdue pegboard Test	ET (s)
Kidgell et al. (2013)	11	R M1	C-SOR; L M1	1	0.040	13	Purdue pegboard test	ET (s)
Marquez et al. (2015)	34	R M1; L M1	C-SOR	1	0.029	20	JHFT	ET (s)
Parikh and Cole (2014)	8	L M1	C-SOR	1	0.040	20	Key slot task	ET (ms)
Sohn et al. (2012)	28	R M1 (A+C); L M1	C-SOR	1	0.040	15	JHFT	ET (s)
Tecchio et al. (2010)	44	R M1	R arm	1	0.029	15	Finger tapping	ET (ms)
Waters et al. (2017)	64	Contralateral M1; Ipsilateral M1	Ipsilateral SOR/M1; contralateral M1	2	0.057	25	Finger sequence	ET (s)
Williams et al. (2010)	20	R M1	L M1	1	0.029	40	JHFT	ET (s)
Abdelmoula et al. (2016)	11	L M1	R Shoulder	1.5	0.043	10	Elbow flexion	TTF at 35% of MIVC (Nm)
Kan et al. (2013)	15	R M1	L shoulder	2	0.083	10	Elbow flexion	TTF at 30% of MIVC (Nm)
Oki et al. (2016)	13	R M1	L SOR	1.5	0.043	20	Elbow flexion	TTF at 20% of MIVC
Radel et al. (2017)	22	R PMC; P PFC	HD montage	2	NS	NS	Elbow flexion	TTF at 35% of MIVC (N)
Williams et al. (2013)	18	R M1	C-SOR	1.5	0.043	20	Elbow flexion	TTF at 20% of MIVC (Nm)
Frazer et al. (2016)	14	L M1	C-SOR	2	0.080	20	Wrist flexion	MIVC (Nm)
Frazer et al. (2017)	13	R M1	C-SOR	2	0.080	20	Elbow flexion	1 RM (kg)
Hendy and Kidgell (2013)	20	L M1	C-SOR	2	0.080	20	Wrist extension	1 RM (kg)
Hendy and Kidgell (2014)	10	R M1	C-SOR	2	0.080	20	Wrist extension	1 RM (kg)
Hendy et al. (2015)	16	R M1	C-SOR	1.5	0.060	15	Elbow flexion	1 RM (kg)

*R, right; L, left; A+C, anodal and cathodal montages used; M1, Primary Motor Cortex; C-SOR, Contralateral Supraorbital Region; SMA, Supplementary Motor Area; PMC, Pre-motor Cortex; PFC, Prefrontal Cortex; RT, reaction time; ET, execution time; TTF, time to failure; MIVC, maximal isometric voluntary contraction; 1 RM, 1 repetition maximum; JHFT, Jebsen Hand Function Test*.

A data extraction form was generated in Microsoft Excel for Mac Version 16.19 (Microsoft Corporation, Redmond, WA, USA), and the following data were recorded: author, sample size, anode/cathode location, current intensity, experimental task, and performance outcome measure. Where possible, the first motor assessment following the first single session of stimulation was used as the post-stimulation measurement. Moreover, significant efforts were made to obtain relevant missing data. Specifically, 19 authors were emailed to request further data, of which six responded.

### Quality and Risk of Bias Assessment

Three bias assessment tools were employed to ensure robust evaluation. The quality and the risk of bias of selected articles were independently assessed by two authors (RP and JA). Quality was assessed using the Jadad score (Jadad et al., 1996) and the van Tulder scale (van Tulder et al., 2003). The Cochrane risk of bias tool (Higgins and Green, 2011) was additionally applied to RCTs with assessment of its seven key components. Any disagreement regarding quality or bias assessment was resolved through discussion with a senior author (HA).

### Data Analysis

Outcome measures including reaction time, task completion time, time to failure, and force, were identified to allow statistical pooling of results. For each outcome measure, individual meta-analyses were performed using all relevant data sources regardless of stimulation protocol. However, where comparative studies used a variety of stimulation sites, further subgroup analyses were performed to examine the change in effect size using only anodal motor cortex stimulation (with variable cathodal placement). Pooled incidence and outcome measures were calculated through a random effects model employing an inverse variance Der Simonian Laird meta-analytical methodology (Tan et al., 2016). Study heterogeneity was appraised through the *I*^2^ statistic and meta-analysis was performed in Microsoft Excel for Mac Version 16.19 (Microsoft Corporation, Redmond, WA, USA) and Stata Version 15 (Stata Corp LP, College Station, TX, USA). Where meta-analysis was not possible, narrative review was performed for additional evaluation of relevant literature.

## Results

### Selected Articles

**Figure 1:**
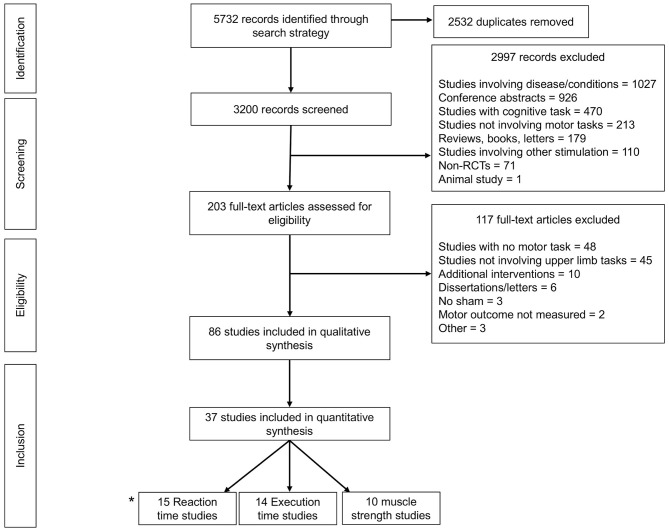
Prisma Flow diagram detailing exclusions throughout each stage of study selection to yield a total of 86 articles for systematic review, 37 of which were meta-analysable. *2 studies (Waters-Metenier et al., 2014; Apšvalka et al., 2018) provided data for both reaction time and execution time.

The flow of articles through the selection process is depicted in Figure 1. Following de-duplication, the literature search yielded 3,200 articles. Following exclusions, 86 relevant articles remained for detailed review. Articles were then subcategorized based on availability of performance outcome data suitable for pooled meta-analysis. These included the following outcome variables: reaction time (RT), execution time (ET), time to task failure (TTF), and force in muscle strength tasks. In total, 37 articles remained for final meta-analysis.

### Overview of Literature

**Figure 2:**
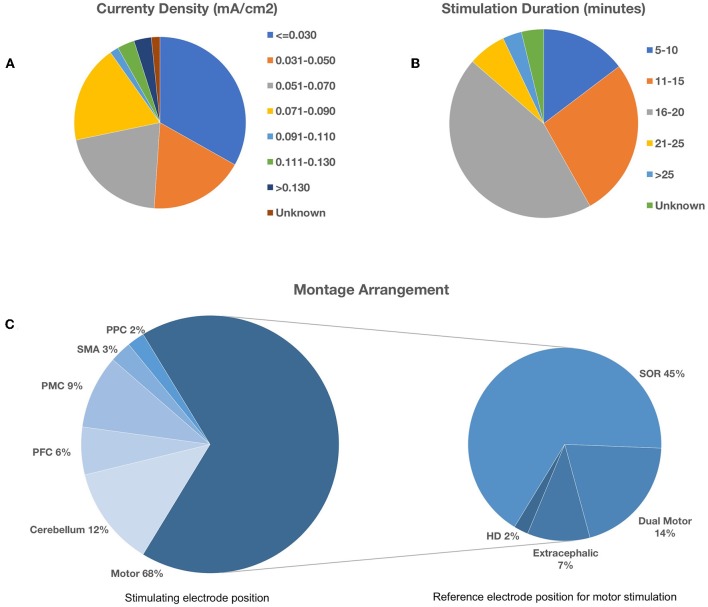
Methodological heterogeneity of selected studies showing variability in **(A)** current density, **(B)** stimulation duration, and **(C)** montage arrangement. Bottom left pie chart illustrates the spread of the target area for stimulation. Bottom right pie chart illustrates the corresponding reference electrode location during motor cortex stimulation. PPC, Posterior Parietal Cortex; SMA, Supplementary Motor Area; PMC, Pre-motor Cortex; PFC, Prefrontal Cortex; SOR, Supraorbital Region; HD, High-Definition.

A total of 86 articles yielded 184 individual montage experiments investigating the impact of tDCS on upper limb motor tasks and there was demonstrable methodological heterogeneity amongst these, as illustrated in Figure 2. The typical stimulation protocol utilized 1 mA with 35 cm^2^ electrodes pads delivering a currently density of 0.029 mA/cm^2^ (30%). Of the total, 43% (*n* = 79) applied stimulation for 20 min and 70% (*n* = 130) used an online approach with motor tasks carried out during the stimulation period. As further illustrated in Figure 2C, motor cortex stimulation was the most frequent target area of choice (67%). There was variability with regard to the montage arrangement within each target area. During motor stimulation, the supraorbital region was the most common (67%) location for the reference electrode.

### Upper Limb Dexterity Tasks—Reaction Time

**Figure 3:**
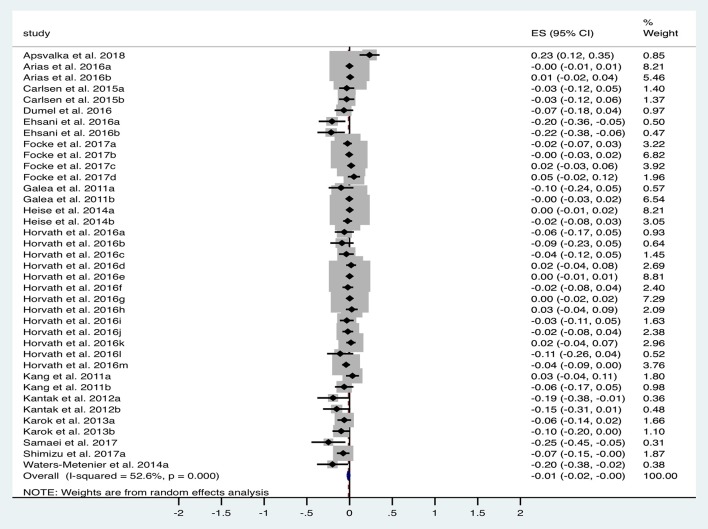
Forest Plot illustrating effect sizes from the comparison in reaction time between tDCS vs. sham. Positive values indicate an increase in reaction time following anodal tDCS whilst negative values indicate a decrease in reaction time. Grey boxes represent the weight given to each study. Error bars represent 95% confidence intervals.

A total of 15 studies (*n* = 618 subjects) were suitable for quantitative analysis of the effect of tDCS vs. sham on RT. As illustrated in Figure 3, tDCS significantly reduced RT, albeit with a small effect size (ES 0.01, 95% CI −0.02 to 0.001, *p* = 0.03). Significant heterogeneity was observed when comparing tDCS to sham (*I*^2^ = 53%; χ^2^ = 78.09, *p* < 0.001). Subgroup analysis of anodal motor stimulation did not alter these results (ES −0.01, 95% CI −0.03 to −0.00, *p* = 0.049). Additional within-group analyses for tDCS and sham groups did not achieve statistical significance. Numerous other studies (summarized in Table 2) investigated the impact of tDCS on RT in a motor task but could not be included in the meta-analysis due to a lack of published raw data. Of these studies, 50% reported improvement with tDCS (80% motor stimulation), which is consistent with the observed marginally beneficial statistical effect size.

**Table 2:** Stimulation protocols and outcomes of additional studies investigating the effect of tDCS on reaction time in an upper limb motor task.

**References**	**Sample size**	**Stimulation**	**Reference**	**Current (mA)**	**Current density (mA/cm^**2**^)**	**Duration (min)**	**Task**	**Significant effect vs. Sham**
Ambrus et al. (2016)	17	L M1 (A+C)	C-SOR	1	0.029	12–14	SRTT	Nil
Dumel et al. (2018)	32	L M1	C-SOR	2	0.044	20	SRTT	↑
Ferrucci et al. (2013)	21	Cerebellum	R arm	2	0.057	20	SRTT	↑
Herzfeld et al. (2014)	51	L M1; Cerebellum (A+C)	C-SOR;R Buccinator	2	0.080	25	Hand reaching	Nil
Leite et al. (2011)	30	L M1, L DLPFC (all A+C)	Right SOR	1	0.029	15	SFTT	Nil
Lindenberg et al. (2013)	20	L M1	C-SOR; R M1	1	0.029	30	Choice RTT	Nil
Lindenberg et al. (2016)	24	L M1	C-SOR; R M1	1	0.029	30	RTT	Nil
Nitsche et al. (2003b)	80	L M1; PMC; L lateral PFC;L medial PFC (all A+C)	C-SOR; R M1	1	0.029	15	SRTT	↑ in L M1
Nitsche et al. (2010)	44	L PMC (A+C)	C-SOR	1	0.029	15	SFTT; SRTT	↑ with A stimulationin REM sleep
Stagg et al. (2011)	22	L M1 (A+C)	C-SOR	1	0.029	15	RTT; SRTT	↑ in A online stimulation;↓ in A/C offline stimulation

*Stimulation sites are anodal unless otherwise specified. R, right; L, left; A, anodal, C, cathodal; M1, Primary Motor Cortex; C-SOR, Contralateral Supraorbital Region; PMC, Pre-motor Cortex; PFC, Prefrontal Cortex; DLPFC, Dorsolateral Prefrontal Cortex; SRTT, Serial Reaction Time Task; SFTT, Serial Finger Tapping Task; ↑, denotes improvement in performance with stimulation; ↓, denotes worse performance with stimulation; Nil, no significant effect of tDCS on performance compared to sham stimulation*.

### Upper Limb Dexterity Tasks—Execution Time

**Figure 4:**
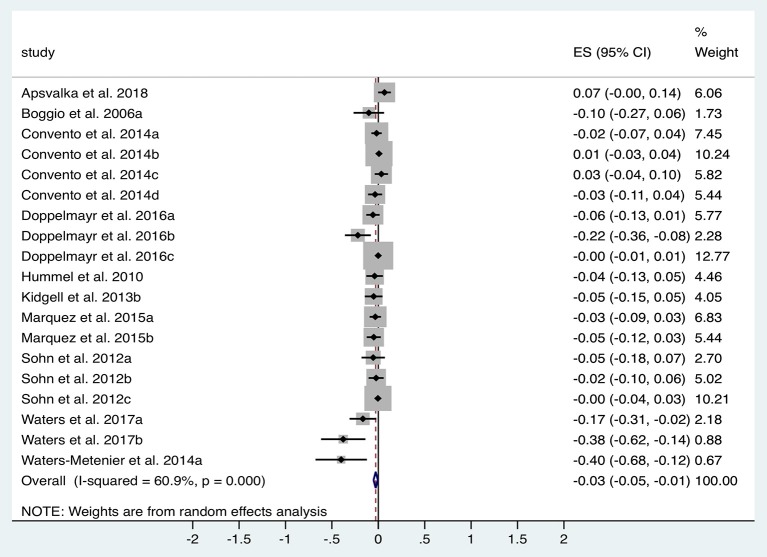
Forest Plot illustrating effect sizes from the comparison in total task time between tDCS vs. sham. Positive values indicate an increase in time taken following anodal tDCS whilst negative values indicate a decrease in time taken. Grey boxes represent the weight given to each study. Error bars represent 95% confidence intervals.

A total of 10 studies (*n* = 344 subjects) were suitable for analysis of the impact of tDCS vs. sham on ET. Figure 4 illustrates the significant reduction in time taken to complete dexterity tasks following tDCS compared to sham with an effect size of −0.03 (95% CI −0.05 to −0.01, *p* = 0.017). Significant heterogeneity was observed (*I*^2^ = 61%; χ^2^ = 46.03, *p* < 0.001). Subgroup analysis of anodal motor montages marginally increased the effect size to −0.04 (95% CI −0.07 to −0.01, *p* = 0.002).

Additional within-group analyses was performed on 11 studies for both tDCS and sham compared to baseline. Overall effect size for tDCS was −0.09 (95% CI −0.13 to −0.05, *p* < 0.001) compared to −0.03 (95% CI −0.05 to −0.004, *p* = 0.02) for sham. Subgroup analysis of anodal motor stimulation confirmed these results for both tDCS (ES −0.09) and in sham (ES −0.02). Additional studies without available data for pooled analysis support overall findings with improved ET in a Purdue Pegboard Test (Karok et al., 2017) and a sport cup stacking task (Pixa et al., 2017a).

### Upper Limb Dexterity Tasks—Accuracy/Error

Numerous studies have explored the impact of tDCS on a series of motor tasks with accuracy and error as outcome measures (Table 3). There is widespread heterogeneity amongst these studies not only in methodological design but also with regard to the task and the definition of the accuracy and error outcome measure. Therefore, we summarize the various montages these and subcategorize them according to the type of outcome measure, namely: correct responses, distance error, degree of error, error count, “skill” (calculated from error and speed measurements of a motor task) and miscellaneous outcome measures.

**Table 3:** Stimulation protocols and outcomes of studies investigating the effect of tDCS on different accuracy and error measurements in motor tasks.

**References**	**Sample size**	**Stimulation**	**Reference**	**Current (mA)**	**Current density (mA/cm^**2**^)**	**Duration (min)**	**Task**	**Significant effect vs. Sham**
**Accuracy: correct responses**								
Dumel et al. (2016)	23	L M1	C-SOR	2	0.044	20	SRRT	Nil
Gomes-Osman and Field-Fote (2013)	28	Bilateral M1	Bilateral SOR	1	0.036	20	SFTT	↑
Karok and Witney (2013)	20	R M1	C-SOR; L M1	1.5	0.060	10	SFTT	Nil
Vines et al. (2008a)	16	R M1	C-SOR; L M1	1	0.061	20	SFTT	↑ in dual motor
Vines et al. (2008b)	17	L M1, R M1 (all A+C)	C-SOR	1	0.061	20	SFTT	↑ L hand in L M1 (C)
Zimerman et al. (2013)	53	L M1	C-SOR	1	NS	20	SFTT	↑ in older subjects
Zimerman et al. (2014)	23	R M1 (C only)	C-SOR	1	0.040	20	SFTT	↓
**Error: distance**								
Doppelmayr et al. (2016)	83	L M1, Cerebellum, R parietal	HD	1	0.318	21	Mirror tracing	Nil
Hardwick and Celnik (2014)	22	L cerebellum	Buccinator	2	0.080	15	Reaching task	↑ in older subjects
Lopez-Alonso et al. (2018)	14	L M1	C-SOR	1	0.040	20	SVIPT	Nil
Matsuo et al. (2011)	14	R M1	C- SOR	1	0.029	20	Circle drawing	↑
Mizuguchi et al. (2018)	24	R Cerebellum (A+C)	R Buccinator	2	0.080	20	Dart throwing	↑ in low performers (C)
Prichard et al. (2014)	54	R M1	C-SOR; L M1	1	0.063	20	Tracing task	↑ in both montages
Taubert et al. (2016)	41	R cerebellum(A+C)	R Buccinator	2	0.080	20	Reaching task	↓ in anodal
Vollmann et al. (2013)	36	L M1, L SMA, L pre-SMA	Forehead	0.75 mA	0.070	20	VPFT	↑ in L M1 + L SMA
**Error: degrees**								
Block and Celnik (2013)	79	L M1; R M1; L cerebellum; R cerebellum	C-SOR; Buccinator	2	0.080	25	VAT	Nil
Galea et al. (2011)	30	L M1; R cerebellum	C-SOR; R Buccinator	2	0.080	15	VAT	↑ in cerebellar
Panouilìeres et al. (2015)	80	L M1; R cerebellum	R SOR	2	0.057	17	VAT	↑ in M1
**Error count**								
Apšvalka et al. (2018)	50	R M1	C-SOR	1	0.029	20	SFTT	Nil
Ehsani et al. (2016)	59	L M1; cerebellum	R SOR; R arm	2	0.080	20	SRTT	↑ in both montages
Horvath et al. (2016)	210	L M1 (A+C)	C-SOR, R M1, R arm	1; 2	0.029; 0.057	20	SRTT	Nil
Leite et al. (2011)	30	L M1, L DLPFC (all A+C)	Right SOR	1	0.029	15	SFTT	Nil
Lindenberg et al. (2013)	20	L M1	C-SOR; R M1	1	0.029	30	Choice RTT	Nil
Lindenberg et al. (2016)	24	L M1	C-SOR; R M1	1	0.029	30	RTT	Nil
Parikh and Cole (2014)	8	L M1	C-SOR	1	0.040	20	Groove pegboard	Nil
Samaei et al. (2017)	30	Cerebellum	R shoulder	2	0.080	20	SRTT	Nil
Shimizu et al. (2017)	45	Cerebellum (A+C)	Buccinator	2	0.057	20	SRTT	Nil
Tecchio et al. (2010)	44	R M1	R arm	1	0.029	15	SFTT	Nil
Vergallito et al. (2018)	24	L PFC; R PFC	C-SOR	1.5	0.060	20	SFTT	↑in L PFC ↑in R PFC in low demand
Waters et al. (2017)	64	Contralateral M1; Ipsilateral M1	Ipsilateral SOR/M1; Contralateral M1	2	0.057	25	SFTT	↑ in both bilateral montages
Waters-Metenier et al. (2014)	52	R M1	L M1	2	0.057	25	SFTT	↑
**Skill: calculated from error and speed**								
Cantarero et al. (2015)	33	Cerebellum (A+C)	R Buccinator	2	0.080	20	SVIPT	↑ in A
Cuypers et al. (2013)	13	L M1	R SOR	1; 1.5	0.040; 0.060	20	SFTT	↑ with 1.5 mA
Hashemirad et al. (2017)	48	L M1; L DLPFC; L PPC	C-SOR	0.3	0.100	20	SVIPT	Nil
Naros et al. (2016)	50	R M1; L M1 (C); R M1; Bilateral M1	C-SOR, C-SOR; L M1; Bilateral SOR	1	0.029	20	Exoskeleton tracing	↑ in all, greatest in bilateral motor
Reis et al. (2009)	36	L M1 (A+C)	C-SOR	1	0.040	20	SVIPT	↑ in both
Rumpf et al. (2017)	47	L M1 (A+C); L PPC	C-SOR	1	0.029	15	SFTT	↑ in L M1 (A)
Saucedo Marquez et al. (2013)	27	R M1	Ipsilateral Shoulder	1	0.040	20	SFTT; SVIPT	↑
Schambra et al. (2011)	87	L M1; R M1	Ipsilateral Shoulder	1	0.040	20	SVIPT	↑ in both. Only L M1 significant
**Miscellaneous**								
Carter et al. (2017)	10	SMA	Forehead	1	0.128	10	Bimanual coordination	↑
Chothia et al. (2016)	12	L Cerebellum	L Buccinator	2	0.125	15	Rotor pursuit	Nil
Ciechanski et al. (2017)	22	L M1	C-SOR	1	0.040	20	Virtual surgical resection	↑
Dumel et al. (2018)	32	L M1	C-SOR	2	0.044	20	Purdue Pegboard	↑
Furuya et al. (2014)	13	R M1; L M1	L M1; R M1	2	0.057	15	SFTT	↑ in both
Goodwill et al. (2013)	11	R M1	C-SOR; L M1	1	0.040	15	VAT	↑
Karok et al. (2017)	30	R M1	C-SOR; L M1	1.5	0.060	15	VPFT	↑ in both montages
Koyama et al. (2015)	28	R M1	L M1	1	0.040	25	Ballistic thumb movements	↑
Lang et al. (2005)	16	L M1 (A+C)	C-SOR	1	0.029	10	SFTT	Nil
Mccambridge et al. (2016)	16	R M1	L M1	1	0.333	15	Circle tracing	Nil
Pixa et al. (2017b)	31	Bilateral M1	HD	1	0.318	15	Purdue pegboard	↑
Rroji et al. (2015)	14	R M1	Ipsilateral shoulder	1	0.040	20	Thumb flexion	↑
Schmidt et al. (2013)	16	Left M1 (C)	C-SOR	0.7	0.020	10	SFTT	↑
Summers et al. (2018)	14	Cerebellum	R buccinator	2	0.029	30	VAT	Nil
Zhu et al. (2015)	27	L DLPFC (C)	C-SOR	1.5	0.060	15-20	Golf putting	↑

*M1, Primary Motor Cortex; SOR, Supraorbital Region; DLPFC, Dorsolateral Prefrontal Cortex; PPC, Posterior Parietal Cortex; HD, High definition; SRTT, Serial Reaction Time Task; SFTT, Serial Finger Tapping Task; SVIPT, Sequential Visual Isometric Pinch Task; VAT, Visuomotor Adaptation Task; VPFT, Visuomotor Pinch Force Task; ↑, denotes improvement in performance with stimulation; ↓, denotes worse performance with stimulation; Nil, no significant effect of tDCS on performance compared to sham stimulation*.

Dual (Vines et al., 2008a; Gomes-Osman and Field-Fote, 2013; Karok and Witney, 2013) and unilateral dominant (Zimerman et al., 2013) motor cortex stimulation increased the *number of correct responses* in a sequential finger tapping task (SFTT), but was not replicated in other studies (Vines et al., 2008b; Dumel et al., 2016). Cathodal stimulation to the non-dominant (Zimerman et al., 2014) motor cortex decreased the number of correct responses in SFTT. tDCS led to improved *skill outcomes*, in the majority of studies applying motor cortex stimulation (Reis et al., 2009; Schambra et al., 2011; Cuypers et al., 2013; Saucedo Marquez et al., 2013; Naros et al., 2016; Rumpf et al., 2017). Similarly, motor stimulation also demonstrated improvements in a variety of miscellaneous tasks (Table 3). Only cerebellar stimulation in this context failed to confer any improvements in motor performance.

Drawing task *distance error* improvements were less consistent with benefits in non-dominant and dual (Matsuo et al., 2011; Prichard et al., 2014), but not dominant motor cortex stimulation (Doppelmayr et al., 2016). Other distance error tasks benefitted with motor (Vollmann et al., 2013) and cerebellar (Hardwick and Celnik, 2014; Mizuguchi et al., 2018) stimulation, but not consistently amongst the literature (Taubert et al., 2016; Lopez-Alonso et al., 2018). Although improvements were demonstrated in visuomotor adaptation tasks (error in degrees) with motor (Panouilìeres et al., 2015) and cerebellar (Galea et al., 2011) stimulation, this was inconsistent (Galea et al., 2011; Block and Celnik, 2013; Panouilìeres et al., 2015). Only a minority of studies (Waters-Metenier et al., 2014; Ehsani et al., 2016; Waters et al., 2017; Vergallito et al., 2018) investigating *error count* in a SRTT and SFTT demonstrated improved performance with tDCS, all of which had substantial variation in stimulation montages.

### Upper Limb Exercise Tasks: Fatigue

**Figure 5:**
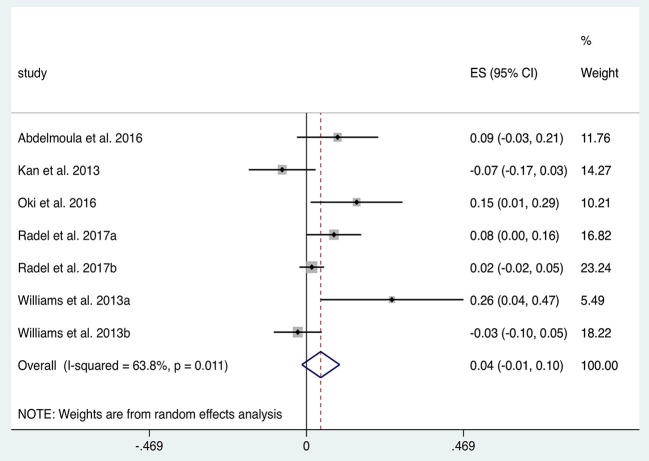
Forest Plot illustrating effect sizes from the comparison in time to elbow flexion task failure between anodal tDCS vs. sham tDCS. Positive values indicate an increase in time to failure following tDCS whilst negative values indicate a decrease in time. Grey boxes represent the weight given to each study. Error bars represent 95% confidence intervals.

In total five studies with *n* = 79 subjects were suitable for quantitative analysis of the effect of tDCS on TTF in elbow flexion tasks. Figure 5 illustrates a tendency towards prolonged TTF with tDCS compared to sham (ES 0.04, 95% CI −0.01 to 0.10, *p* = 0.139). Heterogeneity was observed when comparing anodal tDCS to sham in this cohort of studies (*I*^2^ = 64%; χ^2^ = 16.59, *p* = 0.01). Subgroup analysis of anodal motor montages increased the effect size to 0.06 (95% CI −0.04 to 0.16, *p* = 0.269).

### Upper Limb Exercise Tasks: Strength

**Figure 6:**
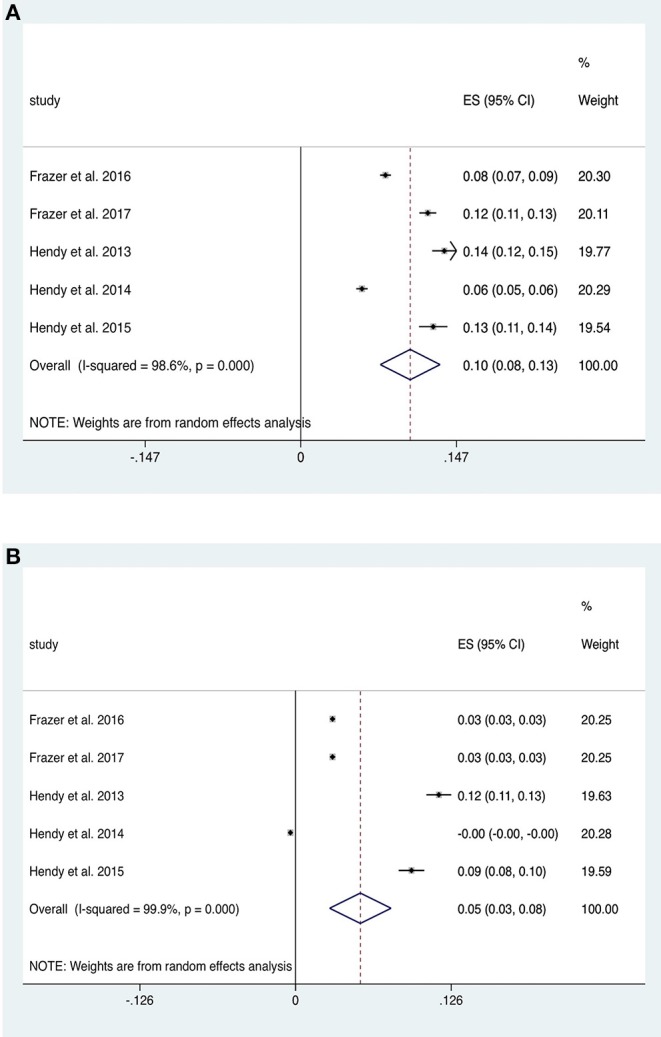
Forest Plot illustrating effect sizes from the comparison in strength between **(A)** anodal tDCS and **(B)** sham tDCS vs. baseline. Positive values indicate an increase strength following each intervention whilst negative values indicate a decrease in strength. Grey boxes represent the weight given to each study. Error bars represent 95% confidence intervals.

Studies investigating the impact of tDCS on strength of contraction in upper limb flexion/extension tasks were divided into four studies with a fatiguing contraction between pre- and post- measurements (therefore causing a decrease in strength) and five studies without such a contraction. The five studies without a fatiguing contraction (*n* = 73 subjects) provided data for within-group analysis of change in strength from baseline in tDCS and sham groups. Anodal motor tDCS increased strength (ES 0.10, 95% CI 0.08 to 0.13, *p* < 0.001; Figure 6A) twice as much as sham (ES 0.05, 95% CI 0.03 to 0.08, *p* < 0.001; Figure 6B). Both of these analyses exhibited significant heterogeneity (*p* < 0.001). A repeated stimulation protocol was utilized in three studies and stimulation was combined alongside strength training (ST) in four studies. An additional study (Lampropoulou and Nowicky, 2013), not included due to lack of data, showed no effect of tDCS on strength.

Elbow flexion strength was examined either side of a fatiguing contraction in four studies. Within-group analyses revealed similar reductions in strength effect size from baseline in intervention (ES −0.26, 95% CI −0.32 to −0.19, *p* < 0.001) and sham groups (ES −0.22, 95% CI −0.28 to −0.17, *p* < 0.001). Subgroup analysis of anodal motor stimulation was comparable.

### Quality Scoring and Risk of Bias Assessment

**Figure 7:**
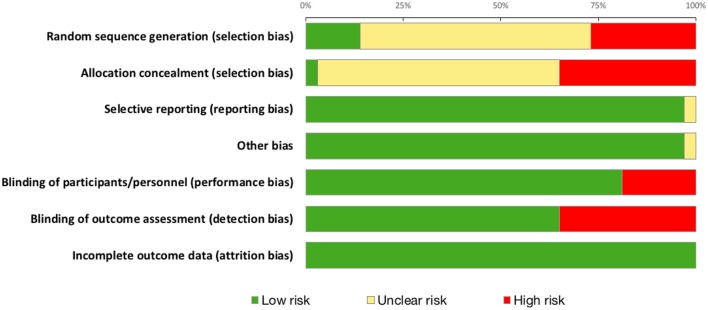
Risk of bias in all 37 studies included for quantitative analysis.

Summary risk of bias graph is illustrated in Figure 7 and Results of Jadad Score and Van Tulder quality assessment scores are summarized in Table 4. Randomization was utilized in 78% of studies but only 14% were deemed to sufficiently explain methods used for random sequence generation. A double-blind approach was used in 65% of studies with the remaining 16% reporting only single-blinding and 19% did not mention blinding at all. Generally, studies performed well in terms of selective reporting, avoiding co-interventions, retaining acceptable compliance and assessing outcomes at similar time-points.

**Table 4:** Total Jadad and Van Tulder studies for each study included in quantitative analysis.

**References**	**Jadad score**	**Van Tulder score**
Apšvalka et al. (2018)	1	7
Arias et al. (2016)	1	5
Carlsen et al. (2015)	0	6
Dumel et al. (2016)	1	6
Ehsani et al. (2016)	5	10
Focke et al. (2017)	3	9
Galea et al. (2011)	3	9
Heise et al. (2014)	3	8
Horvath et al. (2016)	1	5
Kang and Paik (2011)	3	8
Kantak et al. (2012)	1	5
Karok and Witney (2013)	2	6
Samaei et al. (2017)	4	9
Shimizu et al. (2017)	1	6
Waters-Metenier et al. (2014)	3	8
Boggio et al. (2006)	4	9
Convento et al. (2014)	3	8
Doppelmayr et al. (2016)	4	9
Hummel et al. (2010)	3	7
Karok et al. (2017)	2	6
Kidgell et al. (2013)	4	8
Marquez et al. (2015)	5	10
Parikh and Cole (2014)	1	7
Sohn et al. (2012)	3	8
Tecchio et al. (2010)	1	6
Waters et al. (2017)	5	10
Williams et al. (2010)	4	8
Abdelmoula et al. (2016)	1	6
Kan et al. (2013)	1	6
Oki et al. (2016)	3	8
Radel et al. (2017)	4	8
Williams et al. (2013)	4	9
Frazer et al. (2016)	3	8
Frazer et al. (2017)	3	8
Hendy and Kidgell (2013)	4	8
Hendy and Kidgell (2014)	3	8
Hendy et al. (2015)	3	8

*Higher scores represent higher quality*.

## Discussion

This study provides a comprehensive and contemporaneous review and quantitative analysis of the effect of tDCS on in healthy adults. In regard to dexterity tasks, the present analysis has demonstrated a modest improvement in reaction time and significant improvements in execution time and other performance domains of accuracy and error with tDCS. Analysis of muscle strength studies revealed significant strength improvement with training along with a tendency towards reduced fatigue with tDCS.

### Upper Limb Dexterity Tasks

Reduction in motor RT is frequently used as a representation of motor learning, and, numerous studies demonstrate significant reduction in reaction time with tDCS compared to sham. This was commonly observed in unilateral (Nitsche et al., 2003b; Kantak et al., 2012; Karok and Witney, 2013; Heise et al., 2014; Dumel et al., 2016, 2018; Ehsani et al., 2016) and dual (Karok and Witney, 2013; Waters-Metenier et al., 2014) anodal motor stimulation or anodal cerebellar stimulation (Ferrucci et al., 2013; Ehsani et al., 2016; Samaei et al., 2017) with benefits consistent at 24 h retention tests as well (Shimizu et al., 2017). However, improvements were not universal throughout the literature with similar stimulation protocols (Nitsche et al., 2003b; Galea et al., 2011; Stagg et al., 2011; Lindenberg et al., 2013, 2016; Heise et al., 2014; Ambrus et al., 2016; Arias et al., 2016; Horvath et al., 2016; Focke et al., 2017; Apšvalka et al., 2018). Interestingly, RT worsened with cathodal stimulation regardless of site (Leite et al., 2011; Stagg et al., 2011; Carlsen et al., 2015; Shimizu et al., 2017), potentially due to reduced motor cortex excitability with cathodal tDCS (Nitsche et al., 2003a). Further benefits of tDCS in motor tasks was demonstrable with improvements in ET three times greater than sham, a difference made even more apparent when isolating anodal motor stimulation only. All studies with single session anodal stimulation of the non-dominant motor cortex demonstrated improved performance (Boggio et al., 2006; Williams et al., 2010; Sohn et al., 2012; Kidgell et al., 2013; Convento et al., 2014; Parikh and Cole, 2014; Karok et al., 2017). This was not demonstrated with stimulation of the dominant cortex (Boggio et al., 2006; Sohn et al., 2012; Convento et al., 2014) and it is possible that the comparative lack of observed effect on the dominant hand could be due to a ceiling-effect with little room for improvement. However, it could still be beneficial in this context with motor training (Dumel et al., 2018) or in older adults (Hummel et al., 2010). An additional study (Marquez et al., 2015) demonstrated improved performance of the non-dominant hand regardless of laterality of motor cortex stimulation. Amongst other measures of motor performance in dexterity tasks, there is demonstrable and reliable (85% of studies) improvement with dual motor stimulation (Vines et al., 2008a; Gomes-Osman and Field-Fote, 2013; Goodwill et al., 2013; Karok and Witney, 2013; Furuya et al., 2014; Prichard et al., 2014; Waters-Metenier et al., 2014; Koyama et al., 2015; Naros et al., 2016; Karok et al., 2017; Pixa et al., 2017b; Waters et al., 2017). Unilateral motor stimulation was less consistent with as many studies documenting improvement (Matsuo et al., 2011; Reis and Fritsch, 2011; Schambra et al., 2011; Cuypers et al., 2013; Goodwill et al., 2013; Karok and Witney, 2013; Saucedo Marquez et al., 2013; Schmidt et al., 2013; Vollmann et al., 2013; Zimerman et al., 2013; Prichard et al., 2014; Panouilìeres et al., 2015; Rroji et al., 2015; Ehsani et al., 2016; Naros et al., 2016; Rumpf et al., 2017; Dumel et al., 2018) as no effect (Lang et al., 2005; Vines et al., 2008a,b; Tecchio et al., 2010; Leite et al., 2011; Block and Celnik, 2013; Lindenberg et al., 2013, 2016; Parikh and Cole, 2014; Doppelmayr et al., 2016; Dumel et al., 2016; Horvath et al., 2016; Hashemirad et al., 2017; Apšvalka et al., 2018; Lopez-Alonso et al., 2018).

### Upper Limb Exercise Performance

A trend towards increased time to task failure (TTF) with anodal tDCS compared to sham, which was demonstrated in both online and offline stimulation protocols of elbow flexion tasks. The impact of offline tDCS between two fatiguing contractions 1 h apart was examined in three studies (Cogiamanian et al., 2007; Kan et al., 2013; Abdelmoula et al., 2016), two of which (Cogiamanian et al., 2007; Abdelmoula et al., 2016) resulted in improved TTF suggesting potential to help reduce neuromuscular fatigue. Interestingly, all three studies showed no difference between strength (as measured by force) between stimulation and sham. The remaining three studies (Williams et al., 2013; Oki et al., 2016; Radel et al., 2017) utilized an online stimulation protocol, two of which (Williams et al., 2013; Oki et al., 2016) demonstrated an improved TTF. Of note, Williams et al. (2013) performed a subgroup analysis which revealed significantly increased TTF in subjects who had stimulation throughout the task against those who had stimulation for part of the task duration. The former was also found to have worsening strength performance. Although overall there seems to be no consistent effect of tDCS on contraction force when separated by a fatiguing contraction, there does appear to be significantly increased force without such contraction. Indeed, tDCS was found to increase by strength twice as much than sham although it must be noted that this is not a direct comparative analysis. Although methodological variability exists within this pool of studies, separate within-group analyses facilitates a robust comparison of tDCS against sham.

These findings align with a recent meta-analysis by Lattari et al. (2018) on effects of tDCS on upper and lower limb muscle strength which demonstrated improved overall improved muscular endurance (TTF) and strength (force of MVC). More recently, Machado et al. (2019) revealed improved TTF with anodal M1 tDCS in cycling but unlike the present study did not analyse TTF in upper limb tasks. They failed to observe an effect of tDCS on strength in upper limb tasks, although they separated isometric, isokinetic and dynamic upper and lower limb exercises and do not report on three studies (Hendy and Kidgell, 2013; Hendy et al., 2015; Frazer et al., 2017) we included. The current analysis further strengthens the case for the potential of tDCS as an ergogenic aid in tasks requiring muscular endurance and strength, with a potentially more profound impact with training and repeated stimulation.

### Neural Mechanisms

The vast majority of electrode montages in these experiments performed motor cortex stimulation. The mechanism underlying motor learning through tDCS has been postulated as a result of increased excitability of the motor cortex augmenting successful and active synaptic connections between the neuronal structures activated by tDCS (Bindman et al., 1964). This is supported by neurophysiological studies which demonstrate the importance of M1 in early learning (Karni et al., 1995) and also consolidation of learning (Ungerleider et al., 2002; Doyon et al., 2009). However, despite the overall trends for improved motor performance, the evidence is inconsistent. There may be several explanations for these divergent findings. Firstly, there is considerable experimental variation with regard to tDCS parameters (stimulation intensity, duration, anode and cathode placement; see Figure 2), experimental design (e.g., online/offline protocols, timing of motor performance, variable washout periods) and motor tasks and their outcome measures. Secondly, with regards to mechanistic effects, some studies have revealed either minimal change or a decrease in M1 excitability (Jenkins et al., 1994; Toni et al., 1998; Floyer-Lea and Matthews, 2005) suggesting that modulation of this area may not be as influential as previously thought, especially given the large influence of other brain structures in facilitating voluntary movement. Similarly, it is maybe a too simplistic a view to suggest that altering M1 excitability alone will impact on motor learning. Given the well-documented roles of other cortical regions and their interconnections (Doyon et al., 2002; Ungerleider et al., 2002; Hardwick et al., 2013) in performing motor skills, it is perhaps unsurprising that there is such variation in the brain region targeted for stimulation with tDCS. Therefore, it is conceivable that to observe significant gains in motor learning tasks, the reliance on other motor brain areas must be accounted for and augmented as well—a notion which may account for our findings of more consistent improvement with dual motor stimulation (see Table 3). Finally, disparate effects of tDCS may be related to the combination of tasks implemented as slight changes in task can not only affect performance, but also learning processes (Nitsche et al., 2003b; Saucedo Marquez et al., 2013).

Underlying neural mechanisms regarding exercise performance are unclear and a number of factors have been postulated (Cogiamanian et al., 2007). Increases in motor cortex excitability with tDCS (Nitsche and Paulus, 2000, 2001) were not seen in sustained contractions of 20% (Cogiamanian et al., 2007; Williams et al., 2013) and 35% (Abdelmoula et al., 2016) of maximal isometric voluntary contraction (MIVC). However, one of these studies (Williams et al., 2013) did find significant increases in MEPs during a slight contraction following tDCS suggestive of increased cortical excitability. Furthermore, Krishnan et al. (2014) demonstrated increase in EMG magnitude during elbow flexion in higher force levels at 37.5 and 50% of maximum, but not in lower levels. Improvements in force were additionally associated with increased cortical excitability as seen in studies with (Hendy and Kidgell, 2013, 2014; Hendy et al., 2015; Frazer et al., 2017) or without (Frazer et al., 2016) strength training and with (Hendy and Kidgell, 2013; Hendy et al., 2015; Frazer et al., 2016) or without (Hendy and Kidgell, 2014; Frazer et al., 2017) repeated stimulation. These studies also indicate an increase in cross-activation and decrease in short-interval intracortical inhibition as contributory factors. Conversely, other studies have failed to demonstrate MIVC improvement theorized to be due to ceiling effects of maximal muscle contractility (Kan et al., 2013) but also membrane excitability (Williams et al., 2013) as suggested by a lack of difference in MEPs (Lampropoulou and Nowicky, 2013) during elbow flexion.

### Safety Considerations

Given the promising findings in improving upper limb motor performance discussed above, it is important to evaluate the safety aspects neurostimulation technology. Several literature reviews suggest tDCS is safe (Brunoni et al., 2011, 2012; Bikson et al., 2016; Fregni et al., 2016; Woods et al., 2016; Matsumoto and Ugawa, 2017). In an extensive review of tDCS safety (Bikson et al., 2016), no serious adverse events or irreversible injuries were documented in 33,200 sessions in 1,000 subjects including certain potentially vulnerable populations. Common minor side effects include “tingling” and “itching,” which are typically transient and subside following stimulation, and redness, which tends to disappear after 1–2 h. For cumulative exposure, a systematic review (Nikolin et al., 2018) concluded no additional risks to subjects with repeated sessions of tDCS. Healthy subjects have received up to 30 sessions of tDCS without any serious adverse events (Paneri et al., 2015) and some neuropsychiatric patients have received over 100 sessions without any serious adverse events (Andrade, 2013). tDCS has also been shown to be safe in children with over 2,800 sessions on nearly 500 subjects showing no serious adverse effects (Bikson et al., 2016). Two additional reviews also supported these findings with no serious adverse effects observed with tDCS in children (Krishnan et al., 2015; Palm et al., 2016). On a cellular level, Nitsche et al. (2003a) examined neuron specific enolase, a protein associated with neuronal death, in subjects undergoing tDCS and revealed no change in enolase concentration following treatment. In cortical imaging studies, MRI was used to examine subjects for brain oedema, disturbance of the blood-brain barrier and structural alterations of the brain following tDCS and demonstrated no such concerns in any of their subjects (Nitsche et al., 2004). Similarly, Tadini et al. (2011) have confirmed no significant abnormal effects of tDCS on EEG. Furthermore, tDCS is recognised by the National Institute for Health and Care Excellence (NICE) as a safe option in the treatment of depression in adults. It is important to note that this safety profile is assumed only for experiments within certain stimulation protocol limits (e.g., stimulation current up to 2 mA). Although these parameters are being extended (e.g., current up to 4 mA) in ongoing research (Chhatbar et al., 2017), further work is required to ascertain the exact protocol limits for physiological safety.

## Conclusions

The current meta-analysis suggests that tDCS confers immediate performance benefits in dexterity tasks and exercise tasks. Importantly, these results must be interpreted with caution owing to the widespread methodological differences in the experimental domain of tDCS highlighted within this review. Whilst it is appropriate to vary methodology according to the proposed scientific question of the study and also to better appraise the physiological mechanisms of tDCS, the sheer range of methodologies currently utilised has rendered it challenging to group studies for meta-analysis. Additional research is required to delineate neural mechanisms contributing to the effect of tDCS on motor performance which will further our understanding of individual, task and study variability. As the field progresses, narrower stimulation protocols and approaching future work with an emerging standardized manner (Buch et al., 2017) will help to derive more reliable conclusions.

## Limitations

The main limitation of this review lies in the considerable methodological heterogeneity of stimulation protocols, task type and reporting of outcomes. Antal and colleagues (Antal et al., 2015) accurately highlight significant limitations of meta-analysis within the field, some of which are unavoidable due to methodological variability. Accordingly, studies were restricted to those which reported data for the same outcome variable at the same post-stimulation time-point; long-term/retention effects were not within the remit of this study. Similarly, although initial analysis included all protocols to provide an overview of the effect of tDCS, further subgroup analyses of anodal motor stimulation was performed to draw more precise conclusions. Further restricting studies to the same montage, current density and duration would limit available data to an extent that statistical analysis would not be possible or appropriate. Although the present analysis combined single- and multi-session experiments, we deemed this to represent the overall impact of tDCS and where possible, data was extracted after the first session only. Although different tasks were combined for RT and ET analyses, this approach is similar to other published tDCS-related meta-analysis (Dedoncker et al., 2016) and a random-effects model analysis was performed to account for heterogeneity. Finally, individual studies included in the meta-analyses had a small sample size which could potentially reduce the power of analysis.

## Author Contributions

RP, HS, HA, and DL designed the structure and scope of the review. RP, JA, and AP collected review articles. RP prepared the manuscript draft. All authors reviewed and revised the manuscript.

### Conflict of Interest

The authors declare that the research was conducted in the absence of any commercial or financial relationships that could be construed as a potential conflict of interest.

## Appendix 1

### Search Strategy

1. exp transcranial direct current stimulation/
2. (transcranial adj5 electric$ adj5 stimulation).mp. [mp=ti, ab, hw, tn, ot, dm, mf, dv, kw, fx, dq, nm, kf, px, rx, ui, sy, tc, id, tm]
3. (transcranial adj5 DC adj5 stimulation).mp. [mp=ti, ab, hw, tn, ot, dm, mf, dv, kw, fx, dq, nm, kf, px, rx, ui, sy, tc, id, tm]
4. (transcranial adj5 direct current adj5 stimulation).mp. [mp=ti, ab, hw, tn, ot, dm, mf, dv, kw, fx, dq, nm, kf, px, rx, ui, sy, tc, id, tm]
5. tdcs.mp. [mp=ti, ab, hw, tn, ot, dm, mf, dv, kw, fx, dq, nm, kf, px, rx, ui, sy, tc, id, tm]
6. or/1-5
7. Pragmatic Clinical Trial.pt.
8. Randomized Controlled Trial.pt.
9. exp Randomized Controlled Trials as Topic/
10. “Randomized Controlled Trial (topic)”/
11. Randomized Controlled Trial/
12. Randomization/
13. Random Allocation/
14. Double-Blind Method/
15. Double Blind Procedure/
16. Double-Blind Studies/
17. Single-Blind Method/
18. Single Blind Procedure/
19. Single-Blind Studies/
20. Placebos/
21. Placebo/
22. (random* or sham or placebo*).mp.
23. ((singl* or doubl*) adj (blind* or dumm* or mask*)).mp.
24. or/7-23
25. 6 and 24
26. limit 25 to “all adult (18 plus years)”
27. limit 26 to english language
28. limit 27 to human
29. remove duplicates from 28
